# Commensal bacteria education history calibrates the naivety and activation threshold of adaptive antiviral immune system

**DOI:** 10.3389/fimmu.2025.1519023

**Published:** 2025-02-07

**Authors:** Baohua Li, Zhou Sha, Li Mou, Feng Zhang, Lifeng Jia, Yan Zhu, Yan Guo, Guohong Deng, Haibo Wu, Hong Wei, Yuzhang Wu, Lilin Ye, Changjiang Liu, Jian Li, Yanyan Zhang

**Affiliations:** ^1^ Department of Infectious Diseases, Southwest Hospital, Third Military Medical University (Army Medical University), Chongqing, China; ^2^ School of Life Sciences, Chongqing University, Chongqing, China; ^3^ National Health Commission (NHC) Key Laboratory of Birth Defects and Reproductive Health, Chongqing Population and Family Planning Science and Technology Research Institute, Chongqing, China; ^4^ Department of Hepatobiliary Surgery, Tongren Municipal People’s Hospital, Tongren, Guizhou, China; ^5^ Medical Research Center, Chongqing General Hospital, Chongqing University, Chongqing, China; ^6^ Department of Animal Science and Animal Medicine, Huazhong Agricultural University, Wuhan, China; ^7^ Institute of Immunology, Third Military Medical University, Chongqing, China; ^8^ College of Life Sciences, Chongqing College, University of Chinese Academy of Sciences, Chongqing, China; ^9^ School of Biomedical Engineering, Chongqing Medical University, Chongqing, China

**Keywords:** commensal microbiota, adaptive antiviral immunity, germ-free mice, ABX-treated mice, immune education history, T cell immune effect and memory, immune naivety and activation threshold, LCMV (lymphocytic choriomeningitis virus)

## Abstract

Exhaustion of the immune system’s ability to adapt to novelty suggests that the changes it undergoes might be a consequence of an evolutionary unpredictable antigenic exposure over a lifetime. Thus, we raise the question of whether a naive immune system can manage new antigens better than an educated immune system. Here, by employing the naive immune system of germ-free (GF) mice without a history of microbial exposure, we compared their adaptive immune responses with those of the conventional (Conv) mice upon new viral infection. Interestingly, the naive GF immune system showed robust T-cell responses, with more potent memory T cells established for long-term protection, even in the condition of primary lower T-cell levels for naive GF mice. Furthermore, we found that the ABX-treated Conv mice showed impaired T-cell responses, compared with the untreated Conv ones. With the microbiota eliminated, the ABX mice still have a history of microbial exposure and education for their immune system. In summary, commensal bacteria education history calibrates the naivety and the activation threshold of the adaptive antiviral immune system.

## Highlights

- Considering the condition of primary lower T-cell level, the immune system of naive GF mice showed more robust T-cell responses upon LCMV infection.
- With the microbiota eliminated, the ABX-treated mice show impaired T-cell responses, unlike GF mice upon LCMV infection.
- The naive GF immune system shows greater plasticity and sensitivity in launching robust adaptive immune responses against new viral infections.

## Introduction

Adults were thought to have a stronger immunity than children, as they have a more mature immune system with established powerful memory T and B cells against reinfection with experienced pathogens (1). Nevertheless, the clinical course of severe acute respiratory syndrome coronavirus 2 (SARS-CoV-2) infection in children, even in neonates and infants, is milder than that in adults. Children also respond better than adults in the case of other novel viral infections, such as in the 2002 SARS-CoV-1 outbreak, the 2012 MERS-CoV outbreak, and even in the massive 1918 H1N1 influenza pandemic (2–6). Moreover, children also show better effects of vaccination than adults (7, 8). The less‐experienced humoral immunity in children, as evidenced by the higher IgM levels, might induce the production of more potent antibodies upon SARS‐CoV‐2 infection (9). The acute phase of coronavirus disease 2019 (COVID-19) in humans is associated with strong T-cell lymphopenia in severe disease, with a bias toward CD8^+^ T cells (10). Importantly, lymphocyte levels in infected children are not reduced as much as those in adults (9). This central role for T cells makes them a desirable target for assessing immune responses to novel viral infections. The immune system of children is likely prepared to react to novelty in the early years of life to build the pool of memory T and B cells for preventing reinfection, a function that might be dampened in adults.

Exhaustion of the immune system’s ability to adapt to novelty suggests that the changes it undergoes might be a consequence of an evolutionary unpredictable antigenic exposure over a lifetime. From the above, we raise the question of whether a naive immune system is able to manage new antigens better than an educated one. Here, to address the above question, we employed the naive immune system of GF mice without a history of microbial exposure and compared their adaptive immune responses with those of the conventional (Conv) mice (regularly maintained in an SPF condition) at the same age of 6–8 weeks upon new viral infection. For the new viral infection model, we applied the relatively mature LCMV (*lymphocytic choriomeningitis virus*)-P14 CD8 or LCMV-SMARTA CD4 T-cell response system, which can help us to identify the LCMV-specific P14 CD8 (transgenic TCR that is specific for the MHC class I molecule H2-Db-gp33-41 peptide) T-cell responses or the LCMV-specific SMARTA CD4 (transgenic TCR that is specific for the MHC class II molecule H2-I-Ab-gp61-80 peptide) T-cell responses, respectively (11). LCMV is an ideal model to study adaptive immune response, and T- and B-cell responses to LCMV are sensitive and robust, which allows investigators to assess T- and B-cell responses more accurately (11).

Comparing the condition of primary lower T-cell levels of GF mice compared with that of Conv mice, surprisingly, the naive immune system of GF mice showed more robust T-cell responses once they encountered LCMV infection, also with more potent memory CD8^+^ T cells, especially those of Tcm (central memory) and Trm (resident memory) cells, established for long-term protection. Furthermore, in mice treated with ABX (with antibiotic treatment to abolish the microbiota in Conv mice), we found that they showed impaired T-cell responses, not similar to that of GF mice upon LCMV infection. With the microbiota eliminated, the immune system of ABX mice still showed microbial exposure history and education; in other words, their immune system has been educated and is not that naive. Collectively, the above results suggested that the education history of commensal bacteria calibrates the naivety and activation threshold of the adaptive antiviral immune system. This finding, at least from one standpoint, explains the immune system’s naivety‐related differences in adapting to novel stimuli.

## Results

### Mice without a history of microbial education developed robust adaptive immune responses upon encountering new pathogens

**Figure 1:**
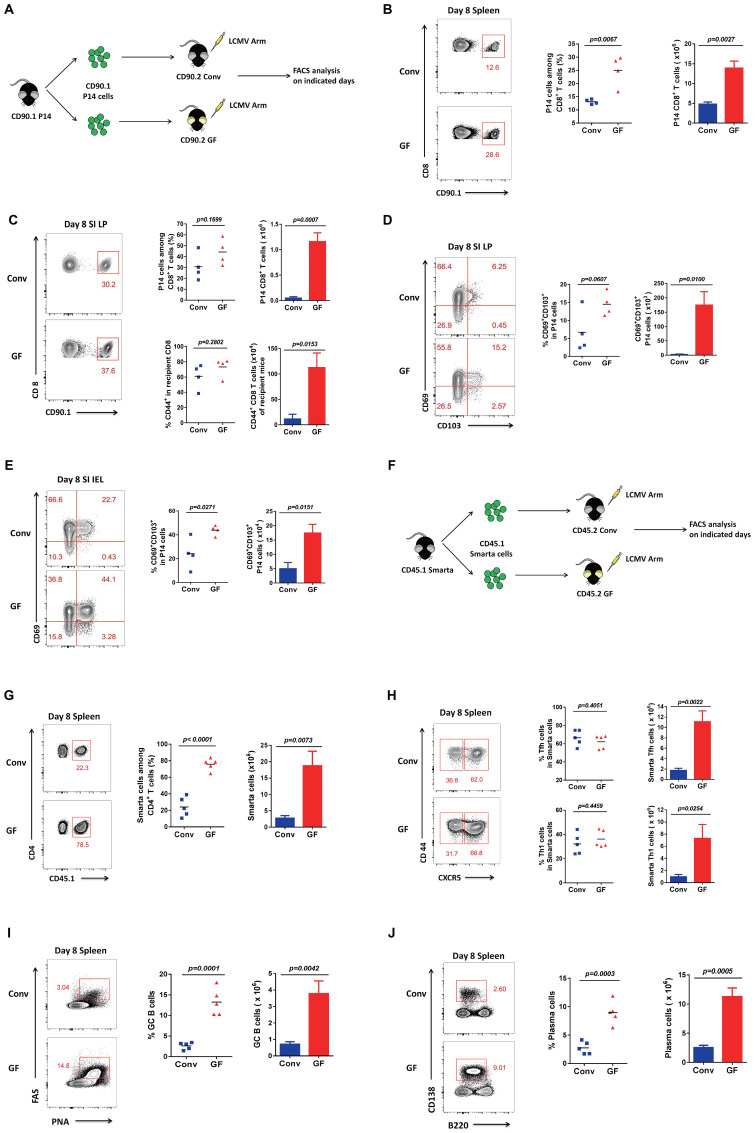
Adaptive immune responses in GF and Conv mice after LCMV infection. **(A)** Experimental design. **(B)** FACS analysis of P14 CD8 T cells among total CD8^+^ T cells of Conv or GF mouse spleens on day 8 post-infection (p.i.). **(C)** FACS analysis of P14 cells among total CD8 T cells or total CD44^+^CD8 T cells among non-P14 CD8 T cells of recipient mice in the SI LP of Conv or GF mice on day 8 p.i. **(D, E)** FACS analysis of CD69^+^CD103^+^ P14 cells (Trm) in the SI IEL or SI LP of Conv or GF mice on day 8 p.i. **(F)** Experimental design. CD45.1 SMARTA cells were adoptively transferred into CD45.2 naive Conv or GF mice following LCMV-Arm infection the next day, and splenocytes were harvested on the indicated days for analysis. **(G)** FACS analysis of CD45.1^+^ SMARTA cells among total CD4 T cells of Conv or GF mouse spleens on day 8 p.i. **(H)** FACS analysis of CD44^+^CXCR5^+^ SMARTA T_FH_ cells or CD44^+^CXCR5^−^ SMARTA T_H_1 cells in the spleen of Conv or GF mice on day 8 p.i. **(I, J)** FACS analysis of PNA^hi^FAS^hi^B220^+^CD19^+^ GC B cells **(I)** or CD138^hi^B220^lo^ plasma cells **(J)** in the spleen of Conv or GF mice on day 8 p.i.

To test whether a naive immune system has a weaker or stronger immunity than an educated immune system upon new viral infection, we studied the immune system of GF mice without a history of microbial exposure and compared their T-cell responses to LCMV infection with those of Conv mice. Firstly, we sorted CD90.1 P14 CD8 T cells from CD90.1 P14 mice and transferred the same quantity of cells into CD90.2 Conv mice or CD90.2 GF mice, respectively, following acute viral infection with the new pathogen LCMV Armstrong (**Figure 1A**). Then, we analyzed the CD8 T-cell responses on the indicated days. Surprisingly, GF mice exhibited a more robust CD8 T-cell immunity than Conv mice, with both the cell proportion and the cell number of the LCMV-specific P14 CD8 T cells elevated dramatically in the spleen on day 8 (**Figure 1B**, **Supplementary Table S1**).

As the microbiota is largely associated with intestinal microbial populations, we also analyzed small intestinal lymphocytes. The intestine contains the largest number of immune cells of any organ in the body, and the intestinal mucosal immune system is composed of three major lymphoid areas: the lamina propria (LP, which lies just underneath the basement membrane in the intestinal villi), the intraepithelial compartment (IEL, which contains intraepithelial lymphocytes and is located between the columnar epithelial cells), and Peyer’s patches (PPs, which are embedded in the gut wall) (12, 13). Here, we first analyzed CD8 T cells in the LP of the small intestine (SI) and found that both the transferred LCMV-specific P14 CD8 T cells and the polyclonal CD44^+^ CD8 T cells of recipient mice were robustly enriched in the GF group compared with those in the Conv group (**Figure 1C**). We also tested CD69^+^CD103^+^ Trm (resident memory T cell) precursors on day 8 in the LP and found that both the cell proportion and the cell number of Trm precursors were overtly increased in GF mice compared with those in Conv mice (**Figure 1D**). We then detected and confirmed that the Trm precursors on day 8 in the IEL also showed a robust increase in both cell proportion and cell number in GF mice compared with those in Conv mice (**Figure 1E**, **Supplementary Figure S1**).

Furthermore, to investigate CD4 T-cell responses, we sorted CD45.1 SMARTA CD4 T cells from CD45.1 SMARTA mice and transferred the same quantity of cells into CD45.2 Conv mice or CD45.2 GF mice, followed by infection with LCMV (**Figure 1F**). Then, we analyzed the CD4 T-cell responses on the indicated days. Consistently, both the cell proportion and the cell number of LCMV-specific SMARTA cells were dramatically augmented in GF mice compared with those in Conv mice (**Figure 1G**), followed by obvious increases in both follicular helper T (Tfh) and T helper 1 (Th1) cell numbers (**Figure 1H**, **Supplementary Figure S3**). Because of the helper functions of Tfh cells for germinal center B-cell (GC B cell) maturation for antibody production, we further detected both germinal center B cells and plasma cells. Likewise, both the cell proportion and the cell number of GC B cells and plasma cells showed a robust increase in GF mice compared with those in Conv mice (**Figures 1I, J**, **Supplementary Figure S4**). These findings demonstrated that mice without a history of microbial education developed robust adaptive immune responses to novel viral infections.

### Mice without a history of microbial education established potent memory CD8 T cells for long-term protection

**Figure 2:**
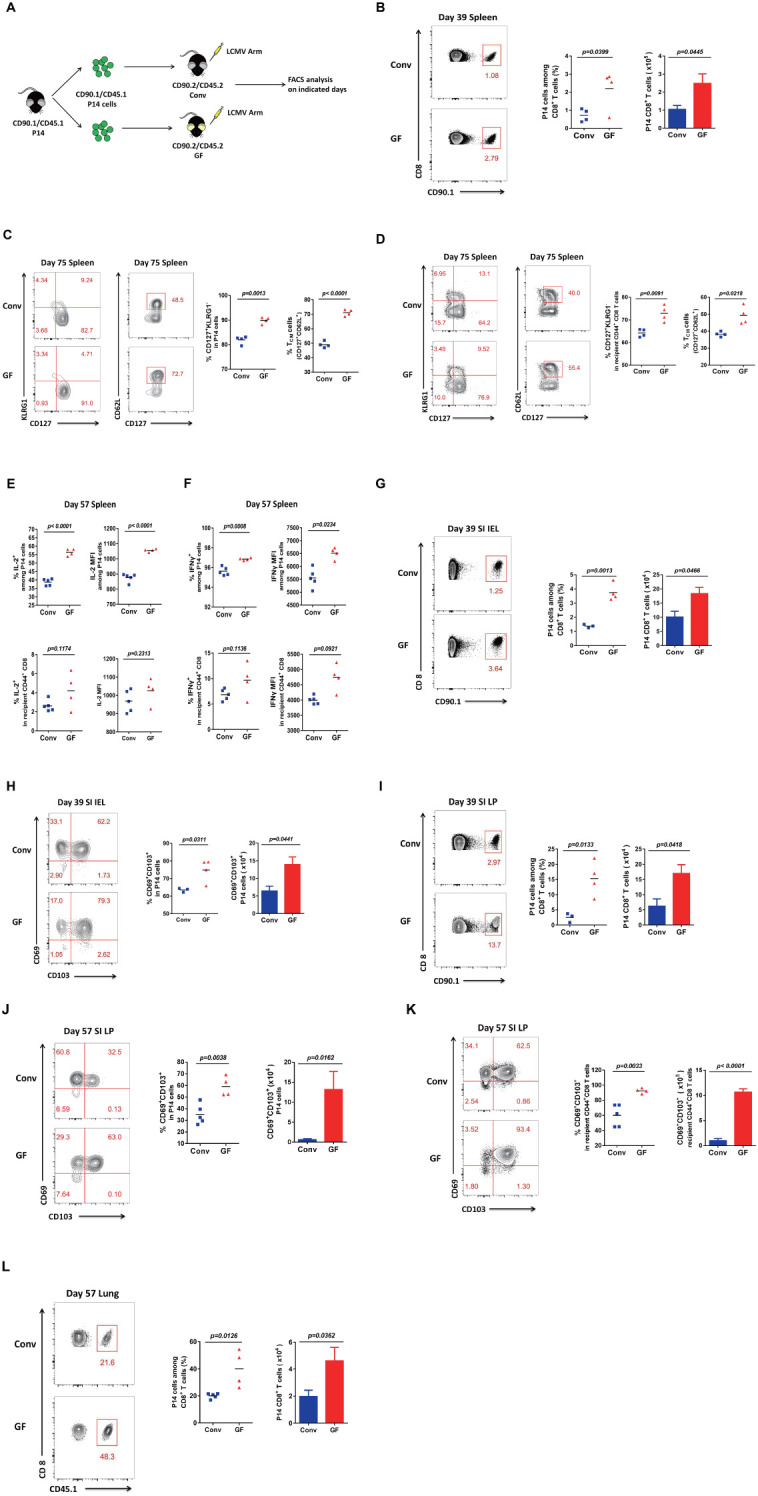
Memory CD8 T-cell responses in GF and Conv mice for long-term protection. **(A)** Experimental design. **(B)** FACS analysis of P14 cells among total CD8 T cells of Conv or GF mouse spleens on day 39 p.i. **(C, D)** FACS analysis of CD127^+^KLRG1^−^ memory or CD127^+^CD62L^+^ Tcm cells among P14 cells **(C)** or among CD44^+^CD8 T cells of recipient mice **(D)** in Conv or GF mouse spleens on day 75 p.i. **(E, F)** The proportion and MFI of IL-2^+^ **(E)** or IFN-γ^+^ **(F)** cells among P14 cells or among CD44^+^CD8 T cells of recipient Conv or GF mouse spleens on day 57 p.i. **(G, H)** FACS analysis of P14 cells **(G)** or CD69^+^CD103^+^ Trm P14 cells **(H)** in the SI IEL of Conv or GF mice on day 39 p.i. **(I, J)** FACS analysis of P14 cells **(I)** or CD69^+^CD103^+^ Trm P14 cells **(J)** in the SI LP of Conv or GF mice on day 39 p.i. **(I)** or on day 57 p.i. **(J)**. **(K)** FACS analysis of CD69^+^CD103^+^ Trm cells among CD44^+^CD8 T cells of recipient mice in the SI LP on day 57 p.i. **(L)** FACS analysis of P14 cells among total CD8 T cells of the lung on day 57 p.i.

To investigate whether mice without a history of microbial education can establish long-term protection, we first sorted CD90.1/CD45.1 P14 CD8 T cells from CD90.1/CD45.1 P14 mice and transferred the same quantity of cells into CD90.2/CD45.2 Conv mice or CD90.2/CD45.2 GF mice, followed by acute viral infection with the new pathogen LCMV Armstrong (**Figure 2A**). Then, we examined LCMV virus-specific P14 CD8 T cells on day 39 in the spleen and found that both the cell proportion and the cell number of P14 CD8 T cells were robustly increased in GF mice compared with those in Conv mice (**Figure 2B**). We further analyzed CD127^+^KLRG1^−^ memory CD8 T cells, especially CD127^+^CD62L^+^ Tcm (central memory T) cells, in P14 cells on day 75 in the spleen. Consistently, not only memory CD8 T cells but also Tcm cells increased in number in GF mice compared with those in Conv mice (**Figure 2C**). We then examined the polyclonal CD127^+^KLRG1^−^ memory CD8 and CD127^+^CD62L^+^ Tcm cells among CD44^+^ CD8 T cells of the recipient mice. The results were consistent with those for the transferred P14 CD8 T cells, with augmentation of both memory CD8 and Tcm cell levels among the polyclonal CD44^+^ CD8 T cells of the recipient GF mice (**Figure 2D**). As laboratory evidence from clinical patients showed greater clonal expansion of T cells with more IL-2 and IFN-γ-producing T cells in moderate disease than in severe disease for COVID-19, we further applied ICC analysis on day 57 in the spleen, testing for IL-2 and IFN-γ, for a deeper understanding of memory CD8 T-cell functions and found that GF mice showed higher IL-2 and IFN-γ levels in both cell proportion and mean fluorescence intensity (MFI) in both P14 CD8 T cells and polyclonal CD44^+^ CD8 T cells of the recipient GF mice compared with those of Conv mice (**Figures 2E, F**, **Supplementary Figure S2**, **Supplementary Table S1**).

We also analyzed small intestinal lymphocytes. First, we found that both the cell proportion and the cell number of LCMV-specific P14 CD8 T cells showed a robust increase on day 39 in the IEL of GF mice compared with those of Conv mice (**Figure 2G**). Furthermore, we analyzed CD69^+^CD103^+^ Trm cells among P14 CD8 T cells and found that Trm cells also showed an obvious increase in both cell frequency and cell number on day 39 in the IEL in GF mice compared with those in Conv mice (**Figure 2H**). We then examined both LCMV-specific P14 cells and Trm cells on day 57 in the LP, and the results were consistent with those for the IEL (**Figures 2I, J**). We further confirmed the robust increase in both the cell frequency and the cell number of Trm cells among polyclonal CD44^+^ CD8 T cells of the recipient GF mice (**Figure 2K**). Finally, to verify the phenotype, we also examined P14 cells in another tissue, the lung, with repeatable results showing a robust increase in both the frequency and the number of P14 cells on day 57 in the lungs of GF mice compared with those of conditional mice (**Figure 2L**). Collectively, the above results showed that mice without a history of microbial exposure established more potent memory CD8 T cells for long-term protection against viral reinfection.

### Mice without a history of microbial education showed primary lower T-cell levels

**Figure 3:**
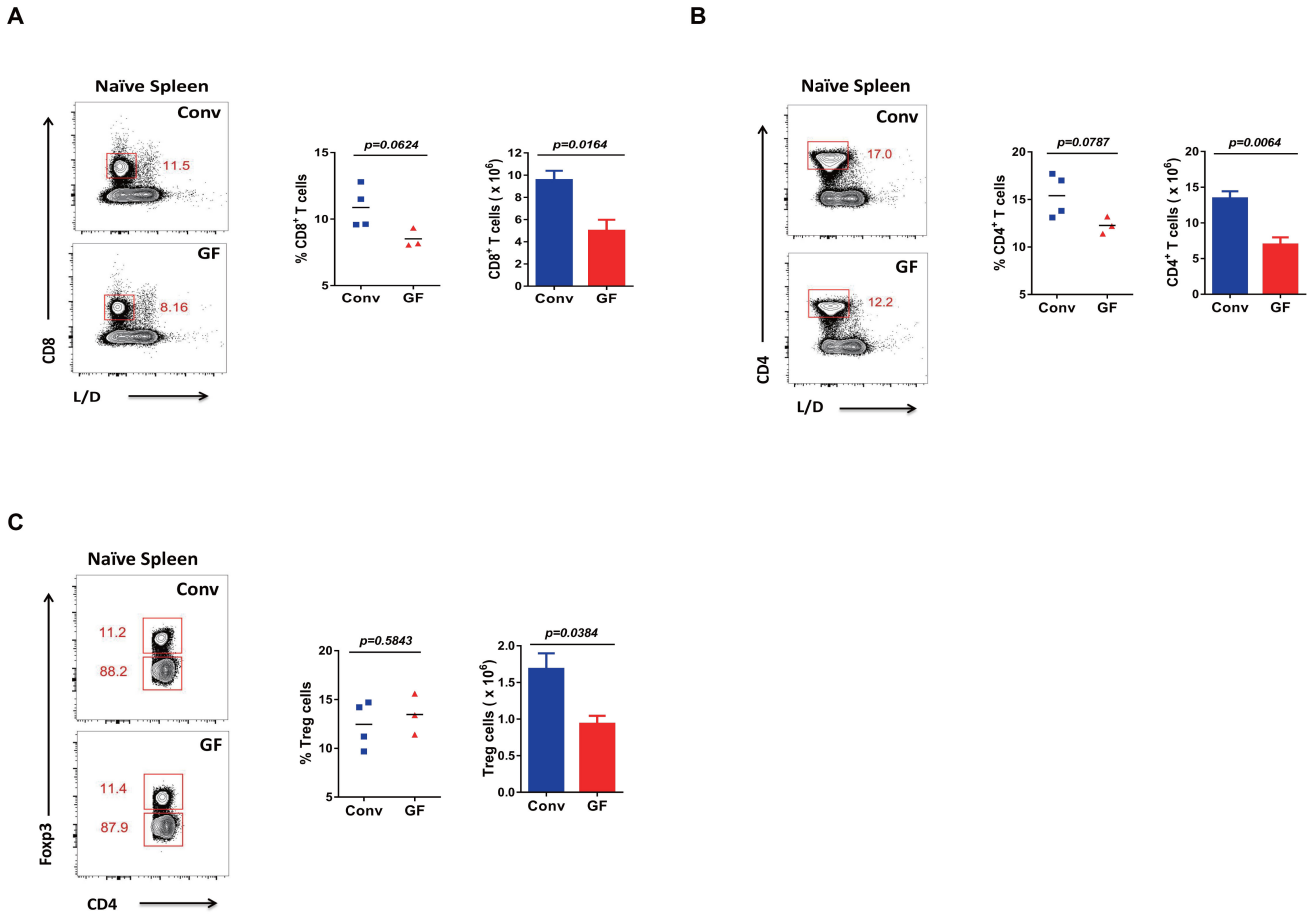
Primary T cell levels in GF and Conv mice. **(A–C)** FACS analysis of L/D^−^CD8^+^ T cells **(A)**, L/D^−^CD4^+^ T cells **(B)**, or L/D^−^Foxp3^+^CD4^+^ Tregs **(C)** in the spleen of naive Conv or GF mice.

Furthermore, we wanted to determine whether naive GF mice have primary lower or higher T-cell levels than Conv mice without LCMV infection. We thus found that both CD8 and CD4 T cells, including regulatory T cells (Tregs with CD4^+^FOXP3^+^), showed a lower primary cell number in naive GF mice than in Conv mice (**Figures 3A–C**, **Supplementary Table S1**).

In short, naive GF mice showed lower levels of primary T cells. This finding was consistent with previous reports (13–15). On the other hand, the above overall results indicated greater plasticity with sensitivity of the naive immune system of GF mice, as shown by the robust adaptive immune responses and immune memory established upon new viral infection, even in the condition of primary lower T cell levels for GF mice compared with that of Conv mice.

### The ABX-treated mice with a history of microbial education exhibited impaired T-cell immunity to new viral infection

**Figure 4:**
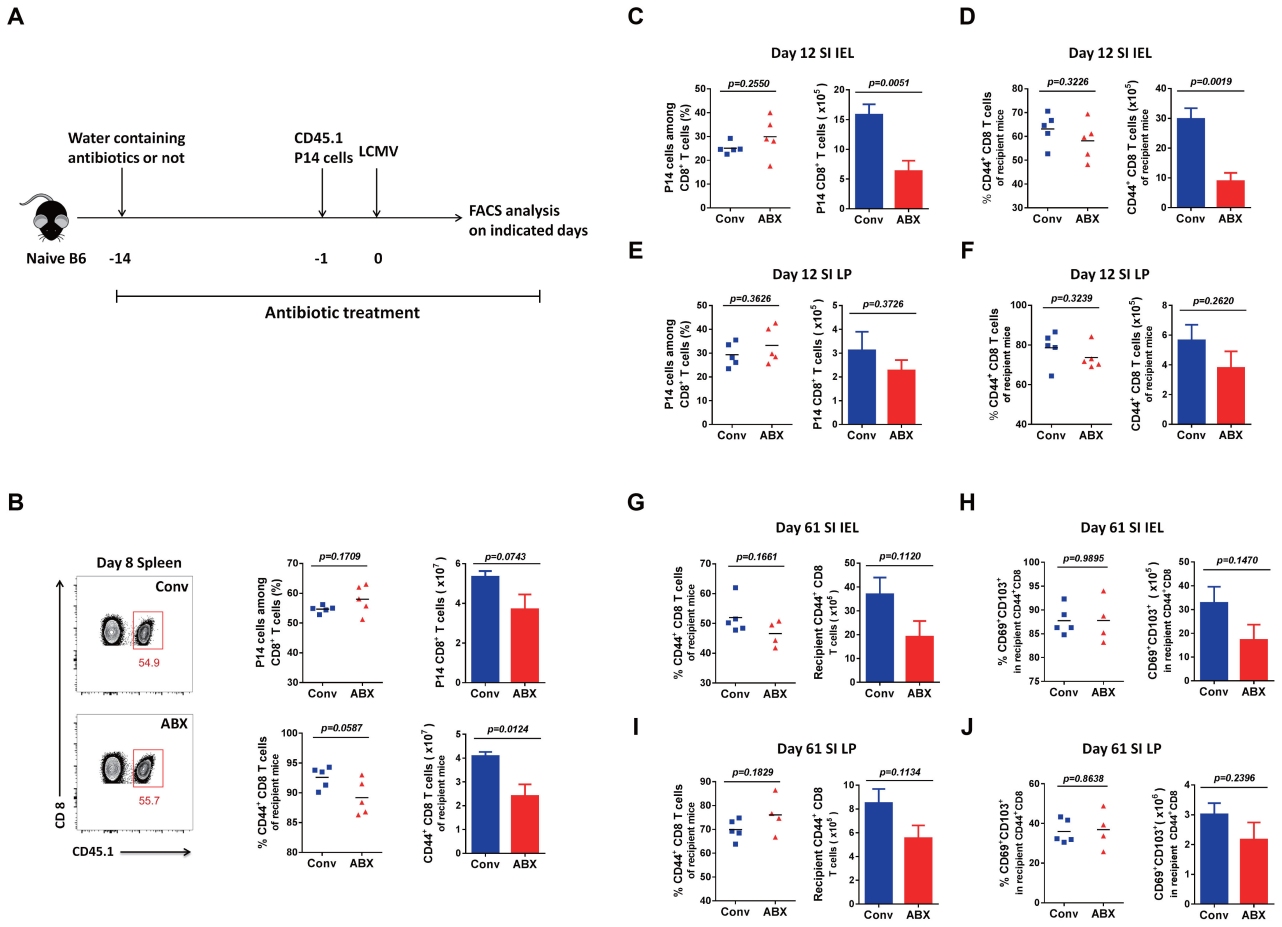
T-cell immunity to LCMV infection in ABX-treated mice and Conv mice. **(A)** Diagram of antibiotic treatment. **(B)** FACS analysis of P14 cells among total CD8 T cells or CD44^+^CD8 T cells among the non-P14 CD8 T cells of recipient mice in the spleen of Conv or ABX-treated mice on day 8 p.i. **(C–F)** FACS analysis of P14 cells among total CD8 T cells or CD44^+^CD8 T cells among the non-P14 CD8 T cells of recipient mice in the SI IEL **(C, D)** or SI LP **(E, F)** of Conv or ABX-treated mice on day 12 p.i. **(G–J)** FACS analysis of CD44^+^CD8 total memory T cells or CD69^+^CD103^+^ Trm cells among CD44^+^CD8 T cells of recipient mice in the SI IEL **(G, H)** or SI LP **(I, J)** of Conv or ABX-treated mice on day 61 p.i.

To investigate whether the microbiota impacts the T-cell responses in Conv mice, we treated mice with antibiotics to eliminate the microbiota (**Figure 4A**). Thus, we compared LCMV-specific P14 CD8 T cells and polyclonal CD44^+^ CD8 T cells of recipients on day 8 in the spleen of the ABX-treated mice with those of normal Conv mice as a control, and we found that both P14 cells and the polyclonal CD8 T cells of the recipient ABX-treated mice showed a decreased cell number (**Figure 4B**). Because of the importance of small intestinal lymphocytes with large intestinal microbiota populations, we also examined P14 cells and polyclonal activated CD8 T cells on day 12 in the IEL and LP, respectively, and found that the results were consistent with those in the spleen; both P14 cells and polyclonal CD8 T cells of the recipient ABX-treated mice exhibited decreased cell numbers in both the IEL and LP (**Figures 4C-F**). Furthermore, we also detected polyclonal memory CD8 T cells and Trm cells for long-term protection on day 61 in the IEL and LP of the recipient ABX-treated mice and Conv mice, with all of them showing a consistently lower number in the ABX-treated mice than in control mice (**Figures 4G–J**, **Supplementary Table S1**).

In brief, with the microbiota eliminated, the ABX-treated mice did not show the same immune responses as the GF mice upon new viral infection, compared with normal Conv mice, respectively. The major difference in their immune system is that the ABX-treated mice still have a history of microbial education; in other words, their immune system is not that naive. Although naive GF mice have fewer primary T cells than Conv mice, they exhibited more robust T-cell responses after new viral infection, including an elevated cell proportion and cell function of memory CD8 T cells for long-term protection. The naive immune system of GF mice with a much abundant naive T-cell repertoire seems to have greater plasticity with sensitivity for aspects of T cells, including T-cell activation, proliferation, differentiation, and function, which is consistent in different organs.

## Discussion

Exhaustion of the immune system’s ability to adapt to novelty suggests that the changes it undergoes might be a consequence of an evolutionary unpredictable antigenic exposure over a lifetime. From the above, we raise the question of whether a naive immune system is able to manage new antigens better than an educated one. Here, by employing the naive immune system of GF mice, we compared the adaptive immune responses of GF mice with those of Conv mice upon LCMV infection. Surprisingly, we found that the naive immune system of GF mice shows robust T-cell responses upon new viral infection, with also more potent memory CD8 T cells established for long-term protection, even in the condition of primary lower T-cell levels for naive GF mice than for Conv ones. Furthermore, we found that the ABX-treated mice showed impaired T-cell responses, unlike GF mice, upon new viral infection. However, even with the microbiota eliminated, the ABX-treated mice still have a history of microbial exposure in their immune system; in other words, their immune system has been educated and is not that naive. In short, the commensal bacteria education history calibrates the naivety and the activation threshold of the adaptive antiviral immune system.

One explanation is that the naive immune system of GF mice also includes both DCs and Tregs that play important roles in T-cell outcomes upon infection. As newly evidenced in the COVID-19 pandemic, the interplay between dendritic cells and CD8 T lymphocytes is a crucial component of SARS-CoV-2 infection immunity (16–18). Moreover, along with the expansion of the memory T-cell pool with continual pathogen exposure, the cell proportion of naive T cells decreases. Under these circumstances, there would be much lower efficiency for DCs to present antigens to the fittest naive T cells. From this perspective, the naive GF immune system with a more abundant naive T-cell repertoire shows greater plasticity and sensitivity to launch more potent T-cell responses upon encountering new viral infection, which at least from one standpoint explains the immune system naivety‐related differences in adapting to novelty.

On the one hand, there are two major classes of Tregs in Conv mice: thymus-derived natural Tregs (nTregs) and intestinally induced Tregs (iTregs) (19). Importantly, iTregs have antigen-specific TCRs for microbiota *in-situ* tolerization, which are different from those of nTregs, and the adoptive transfer of iTregs has been found to affect neither the primary antiviral CD8 T-cell responses nor the viral clearance (20). Moreover, LCMV Armstrong acute infection barely induces iTregs (21). As a result, the T-cell responses that are systemically stronger in the intestines, spleens, and lungs of GF mice than in those of Conv mice are supposedly not due to the effects of Tregs.

Based on the above, we have a suggested perspective on the use of the GF mouse system in intestinal microbiota studies. Compared to GF mice, it would be more appropriate to use ABX-treated mice for research on the impact of intestinal microbiota disruption on immune disturbance. GF mice do not have the same immune starting point as Conv mice. Consistent with previous reports, naive GF mice had primary lower T-cell levels in our study (14), and importantly, their immune system did not have the same history of microbial exposure and education as that of the Conv mice. Regarding humans, there are patients with antibiotic-disrupted microbiota, but there are no GF patients. On the other hand, the GF mouse system is assumed to be more suitable for research on the impact of microbiota colonization on intestinal immune system development.

## Materials and methods

### Mice and virus infection

P14 (CD90.1 or CD45.1) and SMARTA (CD45.1) TCR transgenic mice were obtained from Dr. Rafi Ahmed (Emory University). C57BL/6J (CD45.2) mice were purchased from the Jackson Laboratories. Germ-free mice were provided by Dr. Hong Wei and the Animal Facility of Third Military Medical University, and these GF mice were bred in sterile plastic isolators with autoclaved food and water. All handling procedures for GF mice, including LCMV infection, were carried out under sterile conditions. All mice analyzed were 6–8 weeks of age, and both male and female mice were included without randomization or blinding. LCMV Armstrong was provided by Dr. Rafi Ahmed, and 2 × 10^5^ PFU (i.v.) were used to establish acute infection in mice. Mice infected with LCMV were housed in accordance with Institutional Biosafety Regulations of Third Military Medical University. All experimental protocols were approved by the Institutional Animal Care and Use Committees of the Third Military Medical University. All methods were carried out in accordance with relevant guidelines and regulations that were approved by the Ethics Committees of the Third Military Medical University. This study was carried out in compliance with the ARRIVE guidelines.

### Adoptive cell transfer

In each individual experiment, a total of 2 × 10^4^ naive CD45.1^+^ (or CD90.1^+^) P14 cells or SMARTA cells were adoptively transferred into naive wild-type CD45.2^+^ (or CD90.2^+^) mice, and then these recipient mice were infected with 2 × 10^5^ PFU LCMV Armstrong (i.v.) the next day.

### Lymphocyte isolation from the small intestine and lung

For the isolation of SI IELs, the small intestine and Peyer’s patches were removed, and the intestine was cut longitudinally into 0.5 cm pieces. The intestine pieces were incubated with 0.154 mg/mL of dithioerythritol (DTE) in 10% HBSS/HEPES bicarbonate (30 min at 37°C, 250 rpm) to extract the IEL. For lamina propria lymphocyte (LPL) isolation, gut pieces were further treated with 100 U/mL of type I collagenase (Worthington, Lakewood, USA) in RPMI 1640, 5% FBS, 2 mM of MgCl_2_, and 2 mM of CaCl_2_ for 2 h at 37°C, stirring at 250 rpm. For the isolation of lung lymphocytes, the lung was removed and cut into small pieces, and the pieces were incubated with 1.3 mM of EDTA in HBSS (30 min at 37°C, 250 rpm), followed by treatment with 100 U/mL of type I collagenase (Worthington) in RPMI 1640, 5% FBS, 2 mM of MgCl_2_, and 2 mM of CaCl_2_ (1 h at 37°C, 250 rpm). After enzymatic treatment, lymphocytes were then purified on a 44/67% Percoll gradient (800*g* at 23°C for 20 min).

### Flow cytometry and antibodies

Flow cytometry data were acquired by FACSCantoII (BD Biosciences, Franklin Lakes, USA) and analyzed with the FlowJo software (Tree Star, Ashland, USA). The antibodies and reagents used for flow cytometry staining are listed in **Supplementary Table S1**. Surface staining was performed in PBS containing 2% BSA or FBS (wt/vol). For CXCR5 staining, cells were stained with purified anti-CXCR5 (BD Biosciences) for 1 h at 4°C, followed by biotinylated antirat immunoglobulin G (IgG) (Jackson ImmunoResearch, West Grove, USA) and then fluorescently labeled streptavidin (eBioscience, San Diego, California) for 30 min on ice. Foxp3 staining was performed with the Foxp3/Transcription Factor Staining Buffer Set (eBioscience) after surface staining. For detection of cytokine production, splenocytes were first stimulated with the indicated peptide (0.2 µg/mL), GolgiPlug, GolgiStop, anti-CD107a, and anti-CD107b antibodies (BD Biosciences) at 37°C for 5 h. Following surface staining, intracellular cytokine staining was performed with a Cytofix/Cytoperm Fixation/Permeabilization Kit according to the manufacturer’s instructions.

### Antibiotic treatment

Mice were provided with autoclaved drinking water supplemented with ampicillin (0.5 mg/mL, Solarbio, Beijing, China), gentamicin (0.5 mg/mL, Solarbio), metronidazole (0.5 mg/mL, Solarbio), neomycin (0.5 mg/mL, Solarbio), and vancomycin (0.25 mg/mL, Solarbio). Antibiotic treatment was started 2 weeks before infection and continued for the duration of the experiments.

### Statistical analysis

Statistical analysis was conducted using Prism 6.0 (GraphPad, San Diego, USA). The unpaired two-tailed *t*-test with 95% confidence interval was used for the calculation of *P*-values.

## Data Availability

The raw data supporting the conclusions of this article will be made available by the authors, without undue reservation.
