# Supplementary material for: Commensal bacteria education history calibrates the naivety and activation threshold of adaptive antiviral immune system
Source: Front Immunol. 2025 Feb 7;16:1519023. doi: 10.3389/fimmu.2025.1519023 (PMC11842381; doi:10.3389/fimmu.2025.1519023)
Supplement: Supplementary file 1 [file DataSheet1.pdf]

# Supplementary Materials for

## Commensal Bacteria Education History Calibrate the Naivety and Activation Threshold of Adaptive Antiviral Immune System

Baohua Li[1#], Zhou Sha[2#], Li Mou[3#], Feng Zhang[4], Haibo Wu[2], Hong Wei[5], Yuzhang Wu[6], Lilin Ye[6*], Changjiang Liu[3*], Jian Li[6*] & Yanyan Zhang[7,8,9*]

[1]Department of Infectious Diseases, Southwest Hospital, Third Military Medical University (Army Medical University), Chongqing 400038, China.

[2]School of Life Sciences, Chongqing University, Chongqing 401331, China.

[3]NHC Key Laboratory of Birth Defects and Reproductive Health, Chongqing Population and Family Planning Science and Technology Research Institute, Chongqing, China.

[4]Department of Hepatobiliary Surgery, Tongren Municipal People's Hospital, 554300, Tongren, China

[5]Department of Animal Science and Animal Medicine, Huazhong Agricultural University, Wuhan, China.

[6]Institute of Immunology[PLA], Third Military Medical University, Chongqing 400038, China.

[7]Medical research center, Chongqing General Hospital, Chongqing University, Chongqing 400044, China.

[8]College of Life Sciences, Chongqing College, University of Chinese Academy of Sciences, Chongqing 400714, China.

[9]School of Biomedical Engineering, Chongqing Medical University, Chongqing 400016, China.

***Correspondence**: Yanyan Zhang(yzhangtmmu@163.com), Jian Li(seraph-2004@163.com), Changjiang Liu(cj_514@163.com), Lilin Ye(yelilinlcmv@tmmu.edu.cn)

# Supplementary Figures

**Figure S1. The gating strategy for memory CD8 T cells (MP), Tcm and Trm of P14 .**

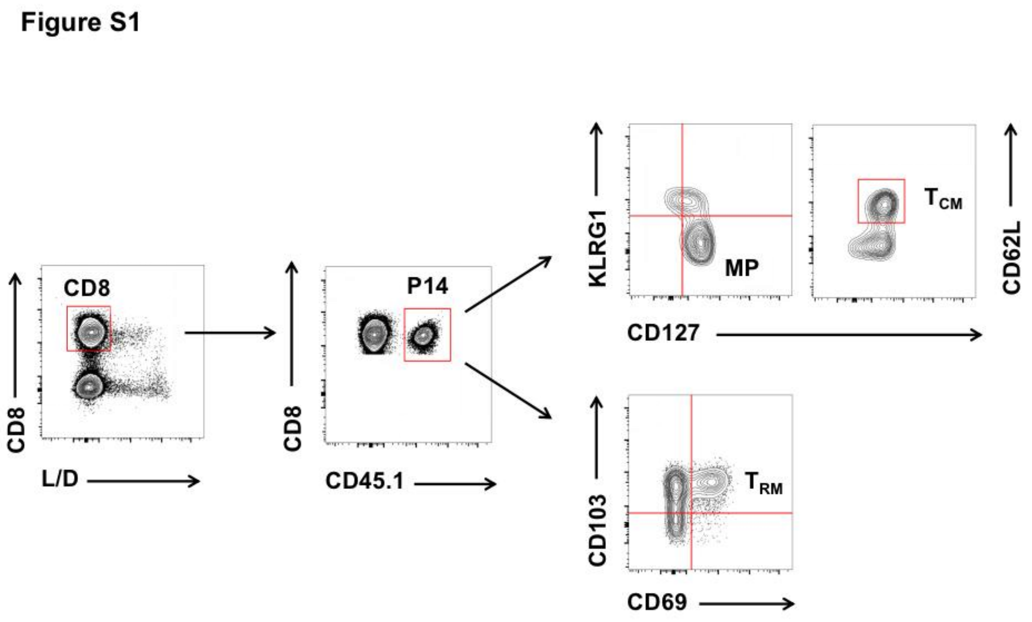

**Figure S2. The gating strategy for Trm of CD8 T cells from recipient mice.**

Figure S2

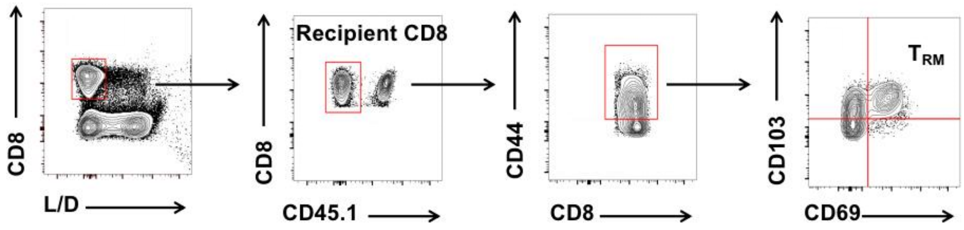

**Figure S3. The gating strategy for Tfh and Th1 cells of CD4 Smarta.**

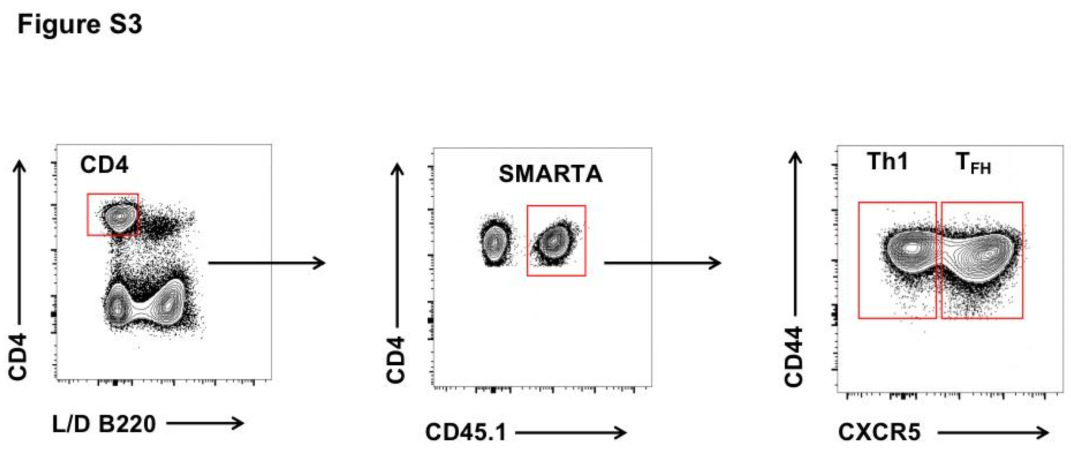

**Figure S4. The gating strategy for Germinal center (GC) B cells and Plasma cells.**

Figure S4

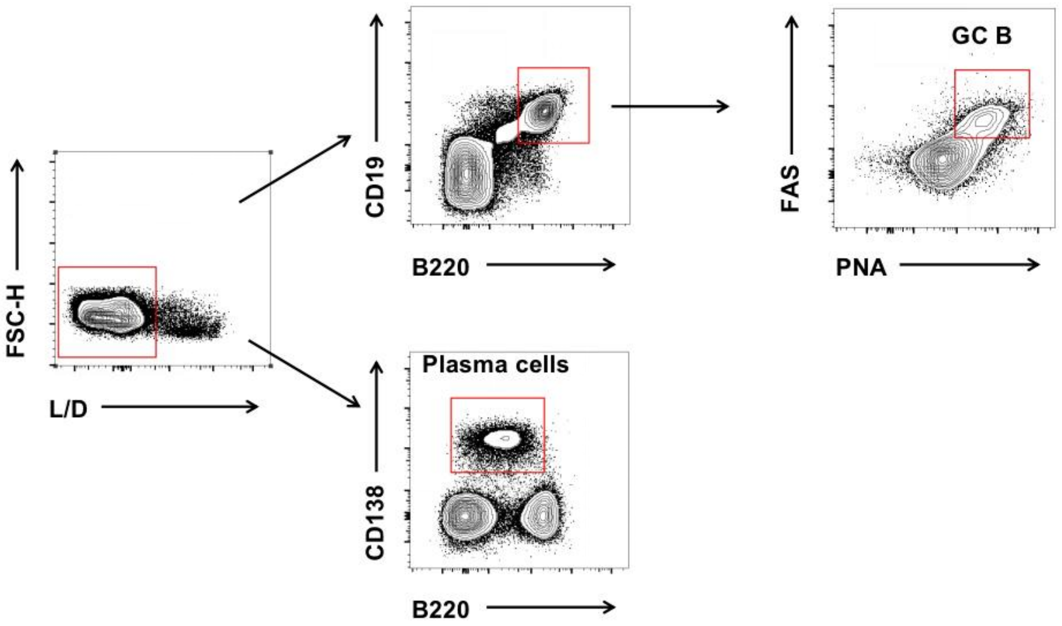

## Supplementary Tables

**Table S1. Antibodies and reagents used in flow cytometry. Related to Figure 1-4.**

| **Antibody/Reagent** | **Cat.#** | **Provider** |
|---|---|---|
| CD4 | RM4-5 | Biolegend |
| CD8 | 53-6.7 | Biolegend |
| CD44 | IM7 | Biolegend |
| CD45.1 | A20 | Biolegend |
| CD90.1 | OX-7 | Biolegend |
| CD62L | MEL-14 | Biolegend |
| CD127 | A7R34 | Biolegend |
| KLRG1 | 138408 | Biolegend |
| Foxp3 | FJK-16s | eBioscience |
| Vα2 | B20.1 | BioLegend |
| CD19 | 6D5 | BioLegend |
| FAS | JO2 | BD Biosciences |
| PNA | FL-1071 | Vector Labs |
| CD138 | 281-2 | BD Biosciences |
| B220 | RA3-6B2 | eBioscience |
| Streptavidin | 25-4317-82 | eBioscience |
| Live/Dead Kit | L10119 | Life Technologies |
| Biotin Goat anti-Rat IgG | 112-065-143 | Jackson Immunoresearch |
| anti-rabbit IgG A647 | 4414S | Cell Signalling Technology |
| Fc-blocker | 2.4G2 | BD Biosciences |
| CXCR5 | 2G8 | BD Biosciences |
| IFN-γ | XMG1.2 | BioLegend |
| CD107a | 1D4B | BD Biosciences |
| CD107b | ABL-93 | BD Biosciences |
| TNF-α | MP6-XT22 | BD Biosciences |
| Granzyme B | GB11 | Invitrogen |
| IL-2 | JES6-5H4 | BioLegend |
